# Risk Scoring System of Mortality and Prediction Model of Hospital Stay for Critically Ill Patients Receiving Parenteral Nutrition

**DOI:** 10.3390/healthcare9070853

**Published:** 2021-07-06

**Authors:** Jee-Yun Kim, Jeong Yee, Tae-Im Park, So-Youn Shin, Man-Ho Ha, Hye-Sun Gwak

**Affiliations:** 1 College of Pharmacy and Graduate School of Pharmaceutical Sciences, Ewha Womans University, 52 Ewhayeodae-gil, Seodaemun-gu, Seoul 03760, Korea; parallelspac@naver.com (J.-Y.K.); jjjhello1@naver.com (J.Y.); 2 Department of Pharmacy, Catholic Kwandong University International St. Mary’s Hospital, Incheon 22711, Korea; phdi@ish.ac.kr; 3 Department of Infectious Disease, Catholic Kwandong University International St. Mary’s Hospital, Incheon 22711, Korea; psycheeeros@ish.ac.kr; 4 Department of General Surgery, Catholic Kwandong University International St. Mary’s Hospital, Incheon 22711, Korea; manho.ha@ish.ac.kr

**Keywords:** intensive care unit, mortality, length of stay, scoring, prediction model

## Abstract

Predicting the clinical progression of intensive care unit (ICU) patients is crucial for survival and prognosis. Therefore, this retrospective study aimed to develop the risk scoring system of mortality and the prediction model of ICU length of stay (LOS) among patients admitted to the ICU. Data from ICU patients aged at least 18 years who received parenteral nutrition support for ≥50% of the daily calorie requirement from February 2014 to January 2018 were collected. In-hospital mortality and log-transformed LOS were analyzed by logistic regression and linear regression, respectively. For calculating risk scores, each coefficient was obtained based on regression model. Of 445 patients, 97 patients died in the ICU; the observed mortality rate was 21.8%. Using logistic regression analysis, APACHE II score (15–29: 1 point, 30 or higher: 2 points), qSOFA score ≥ 2 (2 points), serum albumin level < 3.4 g/dL (1 point), and infectious or respiratory disease (1 point) were incorporated into risk scoring system for mortality; patients with 0, 1, 2–4, and 5–6 points had approximately 10%, 20%, 40%, and 65% risk of death. For LOS, linear regression analysis showed the following prediction equation: log(LOS) = 0.01 × (APACHE II) + 0.04 × (total bilirubin) − 0.09 × (admission diagnosis of gastrointestinal disease or injury, poisoning, or other external cause) + 0.970. Our study provides the mortality risk score and LOS prediction equation. It could help clinicians to identify those at risk and optimize ICU management.

## 1. Introduction

In the era of the COVID-19 pandemic, a large number of patients with respiratory distress or failure are admitted to the hospital and intensive care unit (ICU) [1,2,3]. As the ICU occupancy rate increases, there is a greater need for a prompt diagnosis and an accurate prediction of disease prognosis in the critical care setting [4]. Predicting patients’ prognosis and reducing their length of stay (LOS) in hospitals are beneficial not only for patients themselves, their families, and healthcare professionals but also for the efficient allocation of limited public healthcare resources [5,6,7].

It is well known that patients admitted to the ICU need more hospital resources and intensive care provided by medical staff compared with patients admitted to general wards [8,9]. This can be attributed to the heterogeneous nature of their diseases, including major trauma, burn, major surgery, severe distress in the respiratory system or other organs, and critical infection, which is often presented as sepsis or septic shock [10,11,12,13]. A fast and appropriate medical decision for each patient would increase the chance of recovery from their severe illness, whereas inappropriate treatment could lead to irreversible multiorgan damage or death in serious cases [14]. Hence, predicting clinical progression and providing appropriate treatment are crucial for the survival and prognosis of these critically ill patients [15,16,17,18].

For risk prediction, various statistical approaches have been employed. Statistics is a field of study concerned with the collection, summarization, and analysis of data [19]. Statistical methods are used in every step of medical research ranging from design to implementation [20]. Along with recent progress in machine learning, which develops trained algorithms based on existing data, both traditional statistical methods and machine learning techniques are used to build prediction models in several fields, including healthcare [21]. There also have been studies on ICU mortality risk prediction using statistical and machine learning methods [22,23,24].

Several studies on risk factors for mortality and LOS have been conducted. These studies identified body mass index (BMI), gender, disease severity scores, blood urea nitrogen (BUN), and albumin as risk factors for mortality and/or LOS [3,15,16,25,26,27]. However, these studies are limited by their study population, which included only patients with certain characteristics or disease types, such as liver transplantation, elderly, surgery, or acute respiratory failure. The limitations of previous studies necessitate a better prediction model that includes various disease types reflecting the heterogeneity of patients admitted to the ICU. Therefore, we conducted a retrospective observational study aiming to develop the risk scoring system of mortality and the prediction model of ICU LOS among adult patients admitted to the ICU. By performing this study, it was expected to better understand the prognostic factors of ICU patients and predict their clinical progression by applying risk score models. This study could provide evidence for treatment strategies for ICU patients.

## 2. Materials and Methods

### 2.1. Study Population

A retrospective chart review was conducted using consecutive patients who were at least 18 years of age and who stayed in the ICU receiving parenteral nutrition (PN) for at least 4 days from February 2014 to January 2018 in a hospital. Exclusion criteria were patients with ≥50% of the daily calorie requirement supported by enteral nutrition (EN) or oral intake, malignancy, human immunodeficiency virus (HIV) infection, and duplicate records of staying in medical and surgical ICUs. Data were collected from electronic medical records. This study was approved by the institutional review board of Catholic Kwandong University International St. Mary’s Hospital (approval No. IS17MASI0067) in accordance with the 1964 Helsinki declaration and its later amendments, and the requirement for obtaining informed consent was waived due to the retrospective nature of this study. This study is registered at the Clinical Research Information Service (approval No. KCT0002672).

### 2.2. Data Collection

At the time of admission to the ICU, demographic data including sex, age, weight, and height; diagnosis on ICU admission; discharge status (survival or nonsurvival); comorbidities; Acute Physiology and Chronic Health Evaluation (APACHE) II score; quick Sequential Organ Failure Assessment (qSOFA) score; history of previous surgery; LOS in the ICU; and levels of albumin, creatinine, alanine aminotransferase (ALT), aspartate aminotransferase (AST), and total bilirubin were recorded.

### 2.3. PN Administration

All patients received 2-in-1 (Combiflex, JW Pharmaceutical, Seoul, Korea) or 3-in-1 PN admixtures (Olimel, Baxter, Deerfield, IL, and Winuf, JW Pharmaceutical, Seoul, Korea). Olimel is an olive oil-based PN and Winuf is fish oil-based and enriched with n-3 fatty acid. The composition is shown in Supplementary Table S1. If necessary, additional lipid or protein solution was added to 2-in-1 or 3-in-1 PN admixtures. The composition of TPN was individualized in compliance with guidelines from the American Society for Parenteral and Enteral Nutrition (ASPEN, Silver Spring, MD, USA), the European Society for Parenteral and Enteral Nutrition (ESPEN, Luxembourg), or the Korean Society for Parenteral and Enteral Nutrition (KSPEN, Goyang, Korea).

### 2.4. Statistical Analysis

Continuous variables were compared by Student’s *t*-test. If the variables were not normally distributed as determined by one-sample Kolmogorov–Smirnov and Levene tests, Mann–Whitney test was performed. Chi-square test or Fisher’s exact test was used to compare categorical variables. Univariate and multivariable logistic regression analyses were used to identify risk factors for mortality; the odds ratio (OR) and adjusted OR (AOR) were calculated. Attributable risk was calculated as 1 − (1/AOR). For the length of ICU stay, univariate and multivariable linear regression analyses were used. In the case of skewed data, ICU LOS data were log-transformed for multivariable linear regression analysis. Area under the receiver operating characteristic (AUROC) curve was plotted to determine the cut-off values for predicting mortality. For the scoring system, each coefficient from the logistic regression model was divided by the smallest one and rounded to the nearest integer. All statistical tests were two-sided, and *p* values < 0.05 were considered statistically significant. All statistical analyses were performed using SPSS 20.0 (IBM Corp., Armonk, NY, USA).

## 3. Results

A total of 1179 patients were enrolled, and 734 patients were excluded for the following reasons: staying less than 4 days (*n* = 325), with ≥50% of the daily calorie requirement supported by EN or oral intake (*n* = 223), with cancer or HIV infection (*n* = 181), and with duplicate records of staying in medical and surgical ICUs (*n* = 5). Hence, 445 patients, consisting of 280 males and 165 females, were ultimately included in the analysis.

The mean (SD) age was 64.1 (16.1) years. The average energy and amino acid intake of these patients was 18.2 kcal/kg/day and 3.3 g/kg/day, respectively. The most frequent diagnosis on ICU admission was cardiovascular diseases (*n* = 171, 38%) followed by injury, poisoning, or other external causes (*n* = 82, 18%) and respiratory diseases (*n* = 57, 13%). Table 1 reveals that age, APACHE II score, qSOFA score, and albumin level were significant factors associated with mortality. Among the diagnoses on admission, respiratory and infectious diseases were significant factors associated with mortality.

ROC curve analyses showed that an albumin level of 3.4 g/dL had higher sensitivity (50.5%) and specificity (73.6%) for predicting mortality. The AUROC value for albumin level was 0.667 (95% confidence interval (CI) 0.605–0.728, *p* < 0.001) (Supplementary Figure S1).

As shown in Table 2, neither age nor sex was identified as a significant factor in multivariable analysis using significant factors obtained from univariate analysis. Since infectious and respiratory diseases were significant factors associated with mortality, the two diseases were compared with other diseases. APACHE II score was the significant factor associated with mortality; its AOR was 2.2 for every one-category increase. qSOFA score (≥2) and albumin level (<3.4 g/dL) were also significant factors; their AORs were 2.6 and 1.8, respectively. On ICU admission, patients with respiratory and infectious diseases showed a 2.1-fold higher mortality rate compared with the rate of those without the diseases.

**Figure 1:**
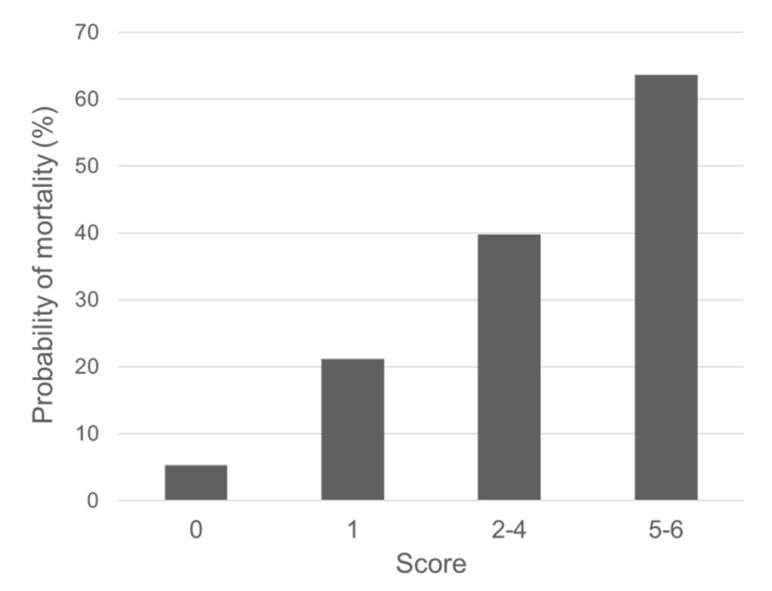
The probability of mortality according to the risk scores.

Based on the aforementioned results, risk scores for mortality were developed (Table 3). APACHE II score (0 points for 0–14, 1 point for 15–19, and 2 points for ≥30), qSOFA score ≥ 2 (2 points), serum albumin level < 3.4 g/dL (1 point), and infectious or respiratory disease (1 point) were incorporated into risk scoring system for mortality. Patients with 0, 1, 2–4, and 5–6 points had approximately 10%, 20%, 40%, and 65% risk of death (Figure 1).

The median ICU LOS was 12 days (range, 4–259 days). Baseline characteristics such as APACHE II score (≥15), albumin level (<3.4 g/dL), AST level (≥40 U/L), ALT level (≥40 U/L), and total bilirubin level (≥2 mg/dL) were associated with longer ICU stay (Table 4). In contrast, patients with gastrointestinal disease or injury, poisoning, or other external cause showed significantly shorter ICU stay.

Multivariable linear regression analysis showed the following prediction equation: log(LOS) = 0.01 × (APACHE II) + 0.04 × (total bilirubin) − 0.09 × (admission diagnosis of gastrointestinal disease or injury, poisoning, or other external cause) + 0.970 (Table 5). Every one-unit increase in APACHE II score and total bilirubin level (mg/dL) increased ICU LOS by 1.0% and 4.0%, respectively; the admission diagnosis of gastrointestinal disease or injury, poisoning, or other external cause was associated with a 9.3% decrease in ICU LOS.

## 4. Discussion

In this study, after adjusting for age and sex, APACHE II score, qSOFA score, serum albumin level, and diagnosis on ICU admission such as respiratory or infectious disease were risk factors for mortality among ICU patients. Higher baseline APACHE II score and serum level of total bilirubin were associated with increased ICU LOS. In contrast, the admission diagnosis of gastrointestinal disease or injury, poisoning, or other external causes was associated with ICU LOS.

There have been several scoring systems to evaluate the severity of ICU patients. Among them, APACHE II/III, Simplified Acute Physiology Score (SAPS) II, and SOFA scores are widely used to predict ICU mortality [28]. According to a systematic review of prognostic scoring systems for ICU care, the proportion of studies that reported very good or good discrimination was 67.7% and 80.8% for APACHE II and SAPS II, respectively [29]. As well as mortality, several studies have investigated the potential predictors for ICU LOS. Verburg et al. reviewed that age, admission source, and comorbidities in addition to severity scores were predictors for ICU LOS [30].

As expected, APACHE II and qSOFA scores, well-known predictors of ICU mortality, were significant factors associated with hospital death in our study population. Our study results are in agreement with the findings of previous studies using the scores of APACHE II and qSOFA as predictors of mortality or LOS for ICU patients [3,15,31].

The serum albumin level on admission has been evaluated as a critical predictor of hospital mortality [18,32]. Corti et al. [33] showed that albumin level <3.5 g/dL was associated with higher mortality risk among patients with a hip fracture. On the other hand, Yin et al. [34] reported serum albumin level <2.92 g/dL as an optimal cut-off value for predicting the mortality of septic patients. In our study, patients with admission serum albumin level <3.4 g/dL were significantly different in their survival probability compared with those with baseline serum albumin level ≥3.4 g/dL.

Total bilirubin level and underlying diseases as predictors of mortality or LOS are rarely investigated. A study using patients with sepsis [35] reported that increased mortality was associated with higher serum total bilirubin level compared with lower serum total bilirubin level (≤1 mg/dL). Another study [36] reported that a higher mean value of total bilirubin level on the second day postoperation was associated with longer LOS in the ICU among patients who had undergone cardiac surgery with extracorporeal circulation. In our study, together with APACHE II score, serum total bilirubin level was significantly associated with ICU LOS. This association of total bilirubin level with ICU LOS may be attributed to the effect of total PN. The patients in this study received PN as their main source of calories; however, the prolonged use of PN might increase the risk of PN-associated liver disease (PNALD), which is characterized by cholestasis and hyperbilirubinemia [37].

There were significant differences in the clinical prognosis of patients with different disease types. Mortality was higher among patients with respiratory or infectious disease compared with patients with other types of disease. This might be attributed to the characteristics of the disease subtypes; acute respiratory failure, pneumonia, severe upper/lower respiratory infection, and sepsis often need invasive respiratory support such as mechanical ventilation or extracorporeal membrane oxygenation. In addition, patients with gastrointestinal disease or injury, poisoning, or other external cause had shorter ICU LOS compared with the ICU LOS of patients without the diseases. This may be attributed to the younger age and healthier condition of patients with injury or poisoning by drugs or biological substances at the time of admission to the ICU. Moreover, it can be inferred that they often present with gastrointestinal symptoms due to the ingestion of drugs or other substances.

In this study, patients who met their daily calorie requirement mainly via EN or oral intake were regarded as those with less severe disease prognosis, such as those with postoperative care following elective surgery and chronic sequelae resulting from acute disease conditions. As we focused on patients with more severe acute diseases, we excluded patients with ≥50% of the daily calorie requirement supported by EN or oral intake based on the definition of significant nutrition feeding by the ASPEN [38].

There are several limitations in our study. First, this study was limited by the retrospective design. Second, we suggested risk scores for mortality and ICU LOS equation using regression models; however, we did not validate the predictability of our study models with other cohorts. Therefore, it remains to be determined whether the prediction equations have any efficacy in predicting the clinical prognosis of ICU patients. Finally, our study excluded younger patients (<18 years) and patients with cancer or HIV infection. Therefore, we could not generalize the results to other ICU settings, which may include pediatric patients, patients with cancer or HIV infection, and patients with good prognosis.

Nevertheless, the strength of our study lies in its large sample size compared with that of previous studies, thus presenting complementary information on the mortality and hospital LOS of patients admitted to the ICU. Specifically, our study population consisting of surgical and medical ICU patients included different disease subtypes such as cardiovascular, gastrointestinal, respiratory, and infectious diseases. The heterogeneous nature of the study population allows us to generalize our study results to other ICU settings.

## 5. Conclusions

This study provides the mortality risk score and LOS prediction equation, which could help clinicians to identify those at risk and optimize ICU management. Our findings indicated that APACHE II score, qSOFA score, serum albumin level, and underlying respiratory or infectious disease were risk factors for mortality and APACHE II score, serum total bilirubin level, and admission diagnosis were associated with ICU LOS. As this study was conducted retrospectively in a single center, a prospective multicenter study is required.

## Figures and Tables

**Table 1:** Association of patient characteristics with hospital mortality.

Characteristics	Total (*n* = 445)	Death (*n* = 97)	Survival (*n* = 348)	*p*
Sex				
Male	280 (62.9)	64 (66.0)	216 (62.1)	0.481
Female	165 (37.1)	33 (34.0)	132 (37.9)	
Age (years)	64.1 ± 16.1	69.2 ± 15.1	62.6 ± 16.1	<0.001
APACHE II	15.1 ± 7.9	19.4 ± 8.4	13.9 ± 7.3	<0.001
qSOFA	0.6 ± 0.7	0.9 ± 0.9	0.5 ± 0.7	<0.001
Body weight (kg)	61.5 ± 13.3	59.9 ± 12.1	62 ± 13.6	0.185
Height (cm)	163.6 ± 9.7	162.6 ± 9.3	163.8 ± 9.8	0.281
BMI (kg/m^2^)	22.9 ± 4.0	22.6 ± 3.8	23 ± 4.0	0.405
Albumin (g/dL)	3.7 ± 0.7	3.3 ± 0.8	3.8 ± 0.7	<0.001
ALT (U/L)	45.4 ± 102.4	48.7 ± 90.1	44.4 ± 105.7	0.718
AST (U/L)	73.5 ± 169.4	87.9 ± 180.5	69.4 ± 166.2	0.341
Creatinine clearance (mL/min)	76.9 ± 47.4	68.7 ± 42.7	79.1 ± 48.4	0.056
Total bilirubin (mg/dL)	0.9 ± 0.9	1.0 ± 1.2	0.9 ± 0.8	0.339
Number of comorbidities	1.5 ± 1.2	1.6 ± 1.2	1.5 ± 1.2	0.232
Previous surgery				
Yes	205 (46.1)	42 (43.3)	163 (46.8)	0.536
No	240 (53.9)	55 (56.7)	185 (53.2)	
EN treatment status ^†^				
Yes	51 (11.5)	14 (14.4)	37 (10.6)	0.299
No	394 (88.5)	83 (85.6)	311 (89.4)	
Types of lipid emulsion				0.510
Olive oil-based	230 (51.7)	53 (54.6)	177 (50.9)	
Fish oil-based	215 (48.3)	44 (45.4)	171 (49.1)	
Admission diagnosis ^‡^				
Cardiovascular disease				0.440
Yes	171 (38.4)	34 (35.1)	137 (39.4)	
No	274 (61.6)	63 (64.9)	211 (60.6)	
Respiratory disease				<0.001
Yes	57 (12.8)	23 (23.7)	34 (9.8)	
No	388 (87.2)	74 (76.3)	314 (90.2)	
Gastrointestinal disease				0.118
Yes	41 (9.2)	5 (5.2)	36 (10.3)	
No	404 (90.8)	92 (94.8)	312 (89.7)	
Infectious disease				0.017
Yes	14 (3.1)	7 (7.2)	7 (2.0)	
No	431 (96.9)	90 (92.8)	341 (98.0)	
Genitourinary disease				0.129
Yes	16 (3.6)	6 (6.2)	10 (2.9)	
No	429 (96.4)	91 (93.8)	338 (97.1)	
Injury, poisoning, or other external cause				0.395
Yes	82 (18.4)	15 (15.5)	67 (19.3)	
No	363 (81.6)	82 (84.5)	281 (80.7)	

Data are presented as number (%) or mean ± SD; ^†^ EN treatment at the first day of ICU; ^‡^ primary or main diagnosis at the first day of ICU.

**Table 2:** Univariate and multivariate regression analyses to identify predictors for mortality.

Predictors	Unadjusted OR (95% CI)	Adjusted OR (95% CI)	Attributable Risk (%)
Male	1.185 (0.739–1.901)		
Age ≥ 65 years	1.901 (1.197–3.019) **		
APACHE II ^a^	2.845 (1.944–4.166) ***	2.197 (1.464–3.297) ***	
qSOFA ≥ 2	3.429 (1.915–6.139) ***	2.604 (1.393–4.869) **	61.6
Albumin < 3.4 g/dL	2.853 (1.783–4.565) ***	1.787 (1.056–3.025) *	44.0
Admission diagnosis of respiratory or infectious disease	3.353 (1.945–5.753) ***	2.053 (1.111–3.793) ***	51.3

Odds ratio (OR); confidence interval (CI); * *p* < 0.05; ** *p* < 0.01; *** *p* < 0.001. ^a^ Every 1-category increase. There were 3 categories for APACHE II (0–14, 15–29, and ≥30).

**Table 3:** Mortality risk scoring system.

Predictors	Beta Coefficient in Logistic Regression Model	Score
APACHE II	0.787	
0–14		0
15–29		1
≥30		2
qSOFA ≥ 2	0.957	2
Albumin < 3.4 g/dL	0.581	1
Admission diagnosis of respiratory or infectious disease	0.719	1
	Total	6

**Table 4:** Association of patient characteristics with ICU length of stay.

Characteristics	*N* (%)	ICU Days (Mean ± SD)	*p*
Sex			0.070
Male	280 (63.0)	21.1 ± 24.0	
Female	165 (37.0)	20.3 ± 25.8	
Age (years)			0.622
≥65	225 (50.5)	19.6 ± 21.7	
<65	220 (49.5)	22.0 ± 27.2	
APACHE II			< 0.001
≥15	201 (45.1)	25.5 ± 29.3	
<15	244 (54.9)	16.9 ± 19.2	
qSOFA			0.204
≥2	57 (12.8)	26.6 ± 38.1	
<2	388 (87.2)	20.0 ± 21.9	
Body weight (kg)			0.699
≥60	254 (57.0)	20.3 ± 25.1	
<60	191 (43.0)	21.4 ± 23.9	
Height (cm)			0.156
≥165	224 (50.4)	21.3 ± 24.7	
<165	221 (49.6)	20.3 ± 24.5	
BMI (kg/m^2^)			0.556
≥18.5	389 (87.4)	20.8 ± 25.3	
<18.5	56 (12.6)	20.7 ± 19.4	
Albumin (g/dL)			0.004
≥3.4	319 (71.7)	19.1 ± 21.4	
<3.4	126 (28.3)	25.1 ± 31.0	
ALT (U/L)			0.027
≥40	113 (25.4)	21.9 ± 20.8	
<40	332 (74.6)	20.4 ± 25.8	
AST (U/L)			0.007
≥40	168 (37.8)	23.6 ± 28.0	
<40	277 (62.2)	19.0 ± 22.2	
Creatinine clearance (mL/min)			0.354
≥30	382 (85.8)	20.3 ± 24.3	
<30	63 (14.2)	23.8 ± 26.2	
Total bilirubin (mg/dL)			0.035
≥2	24 (5.5)	36.1 ± 53.1	
<2	421 (94.5)	19.9 ± 21.7	
Number of comorbidities			0.150
≥2	192 (43.1)	23.0 ± 29.3	
<2	253 (56.9)	19.1 ± 20.3	
Previous surgery			0.799
Yes	205 (46.1)	21.1 ± 24.7	
No	240 (53.9)	20.5 ± 24.6	
EN treatment status ^†^			0.762
Yes	51 (11.5)	20.0 ± 22.9	
No	394 (88.5)	20.9 ± 24.9	
Types of lipid emulsion			0.609
Olive oil-based	230 (51.7)	19.1 ± 19.8	
Fish oil-based	215 (48.3)	22.6 ± 28.8	
Admission diagnosis ^‡^			
Cardiovascular disease			0.554
Yes	171 (38.4)	19.8 ± 19.6	
No	274 (61.6)	21.4 ± 27.3	
Respiratory disease			0.088
Yes	57 (12.8)	20.2 ± 16.4	
No	388 (87.2)	20.9 ± 25.6	
Gastrointestinal disease			0.022
Yes	41 (9.2)	14.6 ± 16.6	
No	404 (90.8)	21.4 ± 25.2	
Infectious disease			0.346
Yes	14 (3.1)	20.9 ± 14.7	
No	431 (96.9)	20.8 ± 24.9	
Genitourinary disease			0.712
Yes	16 (3.6)	19.4 ± 17.7	
No	429 (96.4)	20.8 ± 24.9	
Injury, poisoning, or other external cause			0.024
Yes	82 (18.4)	18.1 ± 23.1	
No	363 (81.6)	21.4 ± 24.9	

^†^ EN treatment at the first day of ICU; ^‡^ primary or main diagnosis at the first day of ICU.

**Table 5:** Multiple linear regression analysis to identify predictors for ICU length of stay.

Predictors	Coefficient (SE)	t	*p*
Intercept	0.970 (0.045)	21.633	<0.001
APACHE II	0.010 (0.002)	4.543	<0.001
Total bilirubin (mg/dL)	0.040 (0.019)	2.146	0.032
Admission diagnosis of gastrointestinal disease or injury, poisoning, or other external cause	−0.093 (0.039)	−2.366	0.018

A multiple regression analysis of log-transformed ICU length of stay was carried out with sex; age; APACHE II score; serum levels of total bilirubin, albumin, AST, and ALT; and admission diagnosis. Standard error (SE).

## Data Availability

Data will be available on reasonable request.

## Supplementary Materials

The following are available online at https://www.mdpi.com/article/10.3390/healthcare9070853/s1, Table S1: Composition of 2-in-1 or 3-in-1 parenteral nutrition admixtures used in this study, Figure S1: Area under the curve for (-) albumin level.

## Abbreviations

intensive care unit (ICU); length of stay (LOS); body mass index (BMI); blood urea nitrogen (BUN); parenteral nutrition (PN); enteral nutrition (EN); human immunodeficiency virus (HIV); Acute Physiology and Chronic Health Evaluation (APACHE); quick Sequential Organ Failure Assessment (qSOFA); alanine aminotransferase (ALT); aspartate aminotransferase (AST); American Society for Parenteral and Enteral Nutrition (ASPEN); European Society for Parenteral and Enteral Nutrition (ESPEN); Korean Society for Parenteral and Enteral Nutrition (KSPEN); odds ratio (OR); adjusted OR (AOR); area under the receiver operating characteristic (AUROC); confidence interval (CI); Simplified Acute Physiology Score (SAPS); parenteral nutrition-associated liver disease (PNALD).
