# Supplementary material for: Risk Scoring System of Mortality and Prediction Model of Hospital Stay for Critically Ill Patients Receiving Parenteral Nutrition
Source: Healthcare (Basel). 2021 Jul 6;9(7):853. doi: 10.3390/healthcare9070853 (PMC8303977; doi:10.3390/healthcare9070853)
Supplement: Supplementary file 1 [file healthcare-09-00853-s001.zip › healthcare-1261683-supplementary.pdf]

Table S1. Composition of 2-in-1 or 3-in-1 parenteral nutrition admixtures used in this study.

**Chamber type**	**2-in-1**	**3-in-1**				
	Combiflex®	Olimel®		Winuf®		
Product	1,100 mL (for peripheral)	N9E 1,000 mL (for central)	N4E 1,500 mL (for peripheral)	1,435 mL (for central)	1,085 mL (for peripheral)	2,020 mL (for peripheral)
Glucose (g)	120	110[a]	113[a]	200[b]	84[b]	157[b]
Glucose (kcal)	480	440	450	728	307	572
Amino acids (g)	20.7	57	38	73	34	64
Nitrogen, N (g)	3.4	9	6	11.7	5.5	10.2
Amino acids (kcal)	83	230	150	292	137	254
Lipid (g)	0	40	45	55	31	57
Lipid (kcal)	0	400	450	546	306	570
Lipid content ratio (%w/w)	N/A	Olive oil/Soybean oil = 80/20		Fish oil/Olive oil/MCT[c]/Soybean Oil = 20/25/25/30		
Total energy (kcal)	563	1070	1050	1,600	750	1400
NPC	480	840	900	1,300	615	1145
NPC/N (kcal/g)	140	93	150	111	112	112

MCT: middle chain triglycerides derived from coconut oil; N/A: not available; NPC: non-protein calorie. [a]Anhydrous, [b]Monohydrate.

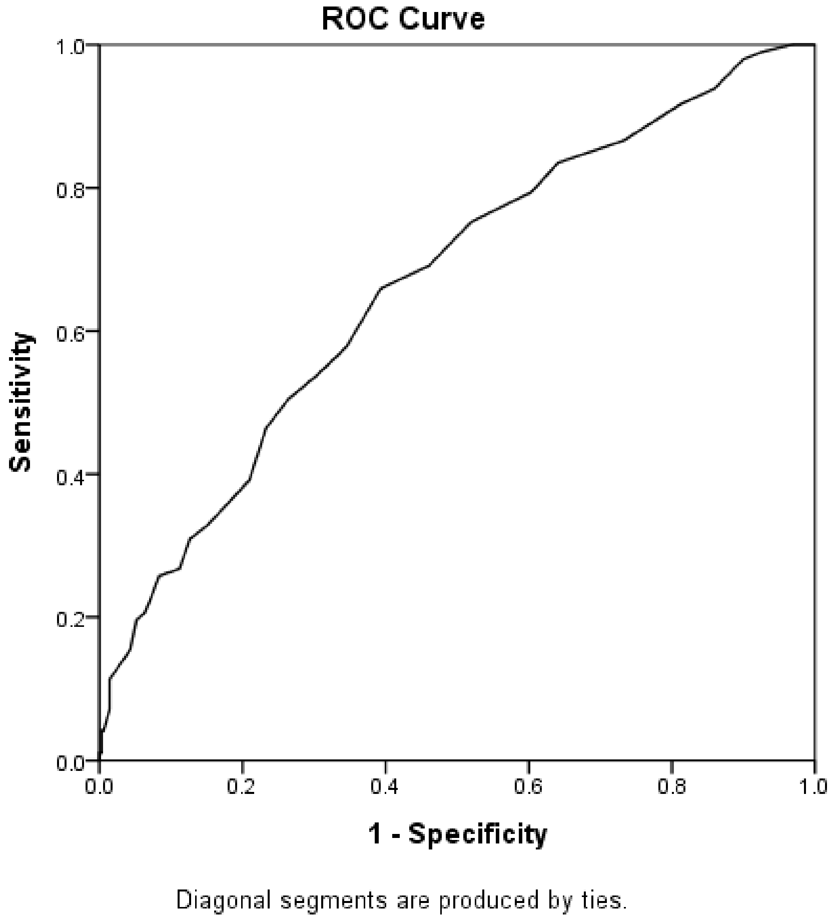

Figure S1. Area under the curve for (-) albumin level.
